# Development of a Novel Method of Spinal Electrophysiological Assessment via Intrathecal Administration at Analgesic Doses

**DOI:** 10.3390/neurolint17050078

**Published:** 2025-05-21

**Authors:** Daisuke Uta, Takuya Yamane, Sosuke Yoneda, Erika Kasai, Toshiaki Kume

**Affiliations:** 1 Department of Applied Pharmacology, Faculty of Pharmaceutical Sciences, University of Toyama, Toyama 930-0194, Toyama, Japan; tkume@pha.u-toyama.ac.jp; 2 Department of Applied Pharmacology, Graduate School of Medicine and Pharmaceutical Sciences, University of Toyama, Toyama 930-0194, Toyama, Japan; takuya.yamane@shionogi.co.jp; 3 Neuroscience, Drug Discovery & Disease Research Laboratory, Shionogi & Co., Ltd., 3-1-1 Futaba-cho, Toyonaka 561-0825, Osaka, Japan; sosuke.yoneda@shionogi.co.jp (S.Y.);

**Keywords:** pain, analgesics, intrathecal administration, electrophysiology, in vivo extracellular recordings, spinal cord

## Abstract

**Background/Objectives**: Chronic pain is a significant global health challenge and is associated with diverse conditions, such as diabetic neuropathic pain and spinal stenosis. Understanding the mechanisms of pain transmission is crucial, for both the peripheral and central pathways. However, there are limitations in spinal electrophysiological techniques in terms of the injection method. Traditional methods such as spinal injections may differ in the distributions and concentrations of drugs compared with intrathecal administration during the behavior test. So, we developed a new intrathecal administration method for electrophysiological recordings. **Methods**: Sprague–Dawley rats were injected with lidocaine intrathecally, and the analgesic effect was evaluated by the von Frey test. In vivo extracellular single-unit recordings of the superficial dorsal horn neurons were performed following a newly developed technique. Lidocaine was intrathecally injected into the arachnoid membrane after laminectomy. After that, the neural responses in the superficial dorsal horn were measured. **Results**: Newly developed intrathecally administered dye reached the spinal cord and the cauda equina. Intrathecally administrated lidocaine increased the paw withdrawal threshold and suppressed spinal neuronal firing. This suppression correlated with increases in paw withdrawal thresholds. **Conclusions**: This innovative method provides insights into the central effects of analgesics, which will help the development of therapies for chronic pain.

## 1. Introduction

Chronic pain represents a significant global health challenge marked by unmet clinical needs regarding its management [1]. Conditions, such as diabetic neuropathic pain resulting from peripheral nerve damage and spinal stenosis and radiculopathy involving injury to central proximal nerves, reflect the diverse causes of chronic pain. This complexity highlights the need for a better understanding of how pain is transmitted and perceived, as unique mechanisms underlie different types of pain [2]. Pain signals follow a well-documented pathway, starting at the peripheral tissue level, where nociceptive stimuli are detected by sensory neurons. These signals are then relayed through peripheral nerves to the spinal cord and subsequently to the brain, where they are processed and recognized as pain [3,4]. This process emphasizes the importance of both peripheral and central structures, including the brain, spinal cord, and proximal nerves, in how we experience pain.

Given the central role of the spinal cord in pain modulation, as well as the significant influence of the peripheral nerves and their projections to the spinal cord, exploring pharmacological interventions that target these areas is crucial [5,6]. Conditions involving damage to both central proximal nerves and peripheral nerves may benefit from targeted drug delivery near the site of injury, potentially maximizing therapeutic efficacy. Recent studies indicated that intrathecal (i.t.) administration of inhibitors targeting peripheral nerve receptors, such as TRPV1 and Nav1.8, can elicit significant analgesic effects [7,8]. Moreover, compounds with high central permeability may exhibit enhanced analgesic properties, emphasizing the importance of both central and peripheral actions [9]. However, understanding the central mechanism remains challenging because of technical limitations. For behavioral studies, i.t. administration is employed to evaluate whether the drug exerts analgesic effects via central nervous system tissues. The i.t. administration involves injecting the drug into the subarachnoid space, which contains cerebrospinal fluid (CSF). As a result, the drug is diluted by the CSF and can diffuse not only into the spinal cord but also into adjacent central nervous structures such as the cauda equina. In contrast, for electrophysiological assessments of the spinal cord aimed at elucidating the underlying mechanisms, spinal injection and bath application methods are used. Spinal injection and bath application deliver the drug directly to the spinal cord, bypassing the CSF, which may result in different drug concentrations within the spinal cord tissue compared to i.t. administration. Therefore, it is inferred that both the concentration of the drug and the extent of its tissue penetration vary depending on the route of administration [10,11]. The differences in dosing regimens make it difficult to precisely determine the spinal neuronal activity associated with the analgesic effects of specific dosages and administration methods.

To address this knowledge gap, we developed a novel i.t. administration method for electrophysiological assessment that reflects the i.t. techniques commonly used in pain behavioral assessment. This approach allows for a clearer understanding of the mechanisms by which analgesics exert their effects. To validate this novel methodology, we aimed to examine the analgesic effects of lidocaine administered via standard i.t. injection and its impact on spinal neuronal inhibition using our newly developed i.t. administration technique. This strategy could not only enhance our understanding of the therapeutic mechanisms involved in pain modulation but could also inform the development of more effective therapeutic strategies.

## 2. Materials and Methods

### 2.1. Animals

Sprague–Dawley male rats aged 5–7 weeks were purchased from CLEA Japan (Tokyo, Japan). The animals were allowed free access to food and water. They were kept in a temperature-regulated room with a 12 h light/dark cycle with lights on at 8:00 a.m. This study was carried out in accordance with Japan’s Animal Welfare and Management Act, the Guide for the Care and Use of Laboratory Animals, and the protocol was approved by the Institutional Animal Care and Use Committee of Shionogi & Co., Ltd., and the University of Toyama.

### 2.2. Intrathecal Administration

For the behavioral assessments, rats were anesthetized with 4% isoflurane and the dorsal skin was shaved. A sterile 26G needle was inserted into the fourth to sixth intervertebral spaces to administer each drug. For in vivo extracellular recordings, rats were anesthetized with urethane (i.p., 1.2–1.5 g/kg) and thoracolumbar laminectomy was performed to expose the L4 to sacral spinal cord region and cauda equina. A sterile 26G needle was inserted into the arachnoid membrane surrounding the cauda equina to inject each drug, 60 min after urethane anesthesia. Lidocaine (final 500 µg/rat; Sigma-Aldrich, St. Louis, MO, USA) was dissolved in saline, and Fast Green (final 0.1%; TCI) was dissolved in artificial cerebrospinal fluid solution. Then both substances were injected intrathecally at a volume of 20 µL. The paw withdrawal threshold and action potentials of spinal neurons were evaluated at 15, 45 and 15, 60, 90 min following the injection of lidocaine, respectively.

### 2.3. Behavioral Tests

The paw withdrawal threshold was assessed using the up–down method [12] with von Frey filaments (vFF) ranging from 1.4 g to 100 g (North Coast Medical, Morgan Hill, CA, USA). Rats were positioned on a mesh floor inside an inverted transparent plastic enclosure. A vFF was gently applied to the central area of the plantar surface of the hind paw, and the withdrawal response was recorded. The lowest force that elicited a withdrawal response was defined as the threshold. Withdrawal thresholds were measured bilaterally and averaged.

### 2.4. In Vivo Extracellular Recordings

In vivo extracellular recordings were conducted as previously described [13]. Briefly, rats were anesthetized using urethane (i.p., 1.2–1.5 g/kg), maintaining consistent anesthesia without the need for further doses. Subsequently, a thoracolumbar laminectomy (L4 to L6) was conducted to expose the L4 to the sacral spinal cord region and the cauda equina. The animals were positioned in a stereotaxic frame, and lidocaine was administered intrathecally. Following a 15 min period to allow for the diffusion of the drug, the dura mater was removed, and the arachnoid membrane was carefully sectioned to create a large opening for the insertion of a tungsten microelectrode into the spinal cord. The exposed spinal cord was continuously perfused with a Krebs solution equilibrated with 95% O_2_ and 5% CO_2_ (10–15 mL/min) containing 2.5 mM CaCl_2_, 1.2 mM NaH_2_PO_4_, 117 mM NaCl, 3.6 mM KCl, 1.2 mM MgCl_2_, 11 mM glucose, and 25 mM NaHCO_3_ at 37 ± 1 °C. Extracellular single-unit recordings were obtained from neurons in laminae I and II of the dorsal horn. Recordings were obtained from neurons located at depths of 20 to 150 µm from the surface. Unit signals were captured using an amplifier (EX1; Dagan Corporation, Minneapolis, MN, USA), digitized through an analog-to-digital converter (Digidata 1400A; Molecular Devices, Union City, CA, USA), and stored on a personal computer using Clampex software (version 10.2; Molecular Devices) for data acquisition and Clampfit software (version 10.2; Molecular Devices) for data analysis. To identify the region on the hind paw that elicited a neural response to a mechanical stimulus, a von Frey filament was applied. A series of von Frey filaments (vFFs; 1.4, 4.0, 8.0, 15.0, and 60.0 g) were tested, and the firing rates of superficial spinal dorsal horn neurons were measured. The stimulus was applied for 10 s at the point of maximum sensitivity in each receptive field of the hindlimb.

### 2.5. Statistics

The data are expressed as mean ± S.E.M. Statistical significance was assessed using Student’s *t*-test for non-paired samples. For multiple comparisons, two-way analysis of variance (ANOVA) was conducted followed by post hoc Sidak’s test. All statistical analyses were carried out using GraphPad Prism version 6.0 (San Diego, CA, USA).

## 3. Results

### 3.1. Development of an Intrathecal Administration Method

**Figure 1:**
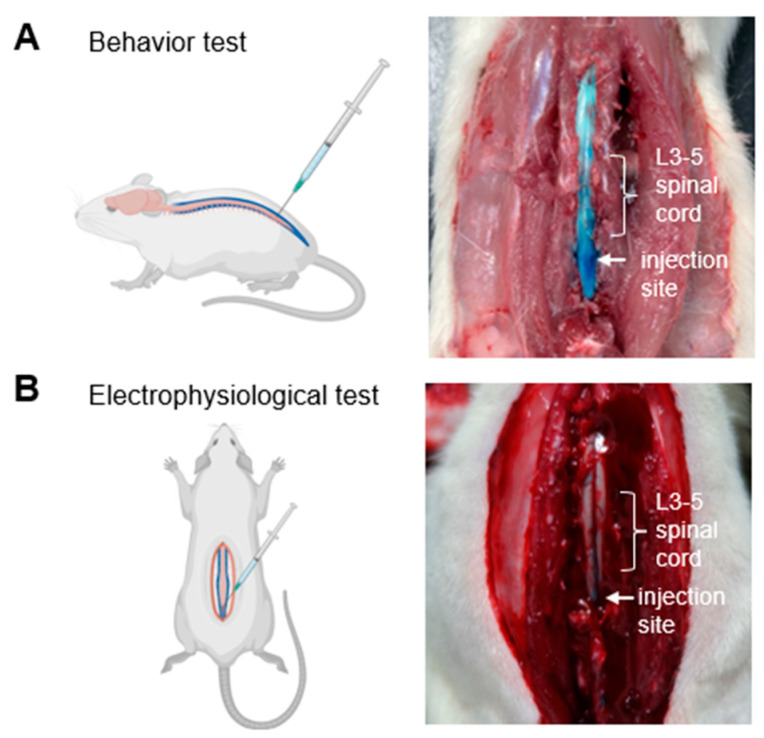
Intrathecal drug delivery and spinal spread during behavioral and electrophysiological assessments. (**A**) Schematic illustration of the intrathecal drug delivery method used during behavioral assessments. The image shows the distribution of the dye (blue). (**B**) Schematic representation of the intrathecal drug delivery method used during electrophysiological assessments. The image shows the spread of the dye. Created with BioRender.com (accessed on 16 April 2025).

First, we developed a novel i.t. administration technique for electrophysiological assessments. For behavioral study, i.t. administration was performed by inserting a needle directly between the vertebrae to deliver the drug solution, avoiding the need for surgical procedures (Figure 1A). In contrast, the spinal electrophysiological evaluations required more extensive preparation, including laminectomy, which significantly increased the pre-assessment time required. Therefore, a laminectomy was performed before drug injection while ensuring the integrity of the arachnoid mater, followed by the delivery of the drug solution via a needle inserted into the subarachnoid space (Figure 1B). To assess the distribution of the drug within central nervous system tissues, we used a dye as a tracer during both procedures. The results indicated that, regardless of the administration technique, the dye effectively spread to the cauda equina and lumbar spinal cord enlargements, confirming adequate distribution to the target areas (Figure 1). These findings suggest that both methods achieve similar levels of penetration within the central tissue, supporting the notion that our newly developed i.t. administration technique is capable of effectively delivering pharmacological agents for subsequent electrophysiological assessments.

### 3.2. Analgesic Effects of Intrathecal Lidocaine

**Figure 2:**
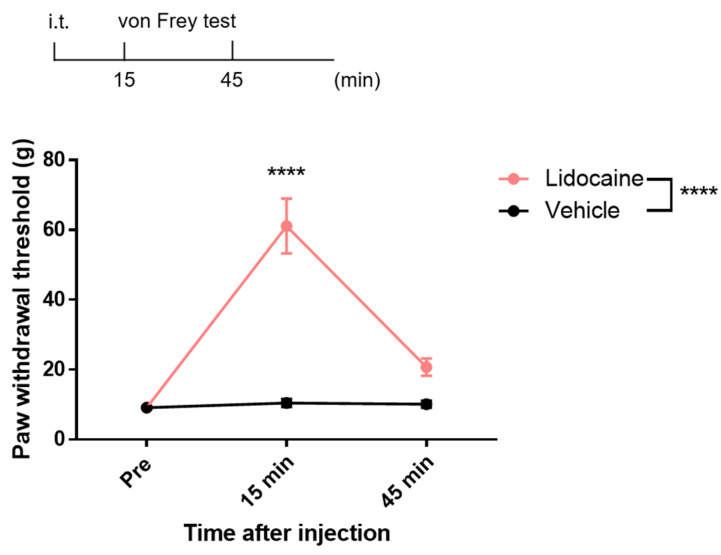
Intrathecal lidocaine administration induced analgesia. Schematic illustration of the time course for the von Frey testing following intrathecal lidocaine administration. Time-dependent changes in the paw withdrawal threshold measured by the von Frey test. Statistical analysis was performed by two-way ANOVA with post-hoc Sidak test. Data are shown as the mean ± standard error (*n* = 8). **** *p* < 0.0001 vs. vehicle.

To evaluate the analgesic effects of i.t. lidocaine and its impact on spinal neuronal activity, we conducted the von Frey test. The results demonstrated that i.t. administration of 0.5 mg of lidocaine significantly increased the paw withdrawal threshold compared to vehicle treatment (Figure 2). Time-course analysis revealed that the peak analgesic effect occurred 15 min post-administration, while a decline in efficacy was observed at 45 min. Nevertheless, the paw withdrawal threshold at this time point remained elevated compared with the vehicle group (Figure 2). These findings indicate that lidocaine is effective between 15 and 45 min after i.t. administration and spinal neuronal activity should be assessed during this time window.

### 3.3. Evaluation of Spinal Neuronal Activity After Intrathecal Lidocaine

**Figure 3:**
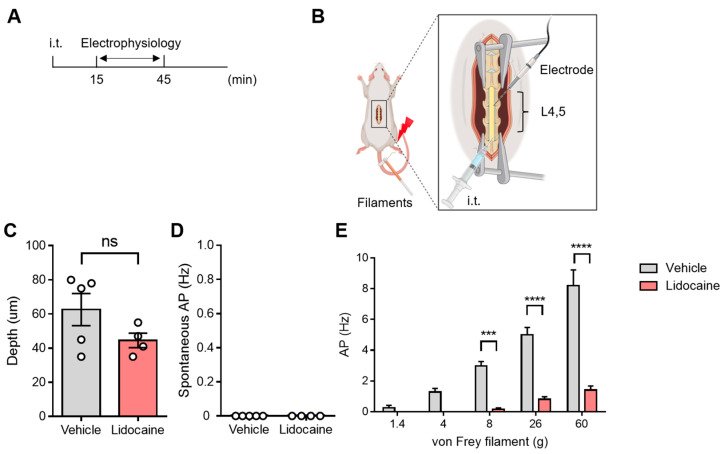
Evaluation of spinal neuronal activity after intrathecal lidocaine. (**A**) Schematic illustration of the time course for electrophysiological evaluation following intrathecal lidocaine administration. (**B**) Schematic illustration of in vivo extracellular recordings. (**C**) The recording depth. (**D**) Spontaneous action potentials. (**E**) Action potentials induced by stimulation of hind paw with von Frey filament. Statistical analysis was performed by unpaired *t*-test (**C**,**D**), two-way ANOVA with post hoc Sidak test (**E**). Data are shown as the mean ± standard error (vehicle n = 5, lidocaine n = 4). *** *p* < 0.001, **** *p* < 0.0001 vs. vehicle. ns: not significant, L4: lumber 4, L5: lumber 5. Created with BioRender.com (accessed on 16 April 2025.).

To assess the feasibility of conducting electrophysiological evaluations of spinal neuronal activity following i.t. administration, we utilized lidocaine and evaluated its analgesic effects using the von Frey test. Following i.t. administration, we applied von Frey stimuli to the plantar surface while recording extracellular single-unit activity from superficial dorsal horn neurons in the L4–5 spinal segments (Figure 3A,B). We found no significant differences in recording depth between the vehicle and lidocaine groups, and spontaneous neuronal firing was not detected in either group (Figure 3C,D). Importantly, the firing frequency of spinal superficial neurons increased in a stimulus intensity-dependent manner, and this response was significantly suppressed after i.t. lidocaine administration (Figure 3E). These results indicate that our electrophysiological assessment method can effectively evaluate spinal neuronal activity after i.t. drug administration. Additionally, the observed suppression of nociceptive transmission following lidocaine administration reinforces its potential role in modulating spinal neuronal responses. These findings highlight the utility of this method for further exploring the central mechanisms involved in pain modulation.

### 3.4. Washout and Long-Term Assessment of Lidocaine

**Figure 4:**
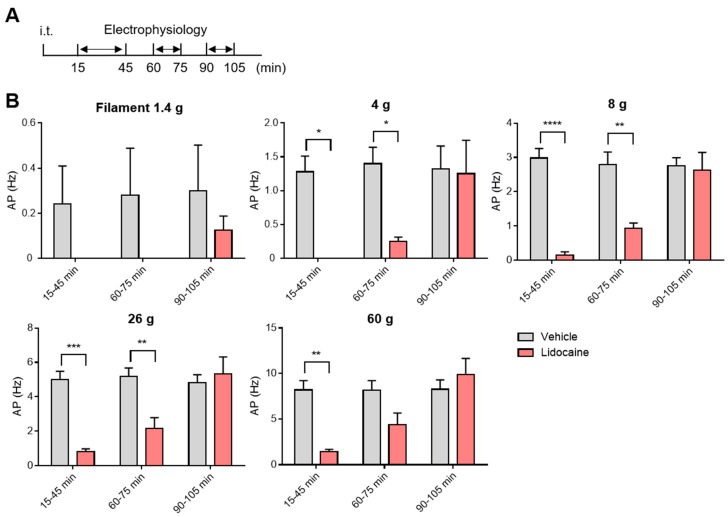
Time-course of spinal neuronal activity after intrathecal lLidocaine. (**A**) Schematic illustration of the time course for electrophysiological evaluation following intrathecal lidocaine administration. (**B**) Action potentials after intrathecal lidocaine administration. Statistical analysis was performed by two-way ANOVA with post hoc Sidak test. Data are shown as the mean ± standard error (vehicle n = 5, lidocaine n = 4). * *p* < 0.05, ** *p* < 0.01, *** *p* < 0.001, **** *p* < 0.0001 vs. vehicle.

To determine whether the suppression of spinal neuronal firing by lidocaine was not attributed to nerve injury caused by the i.t. administration procedure, we conducted evaluations at 15, 60, and 90 min post-administration (Figure 4A). At 15 min, a significant decrease in neuronal firing frequency was observed (Figure 4B). However, this suppression gradually diminished over time, with the effects decreasing notably after 60 min (Figure 4B). By 90 min post-administration, there was no significant difference in spinal neuronal firing frequency between the lidocaine group and the vehicle group, indicating that both groups had returned to similar levels (Figure 4B). Notably, similar results were obtained with full-strength von Frey filaments. These findings suggest that the effects of i.t. lidocaine administration are likely to be caused by transient neuronal activity suppression rather than nerve damage. Furthermore, this method demonstrates potential for evaluating the long-term effect of drugs.

## 4. Discussion

In this study, we developed a novel method that utilizes i.t. administration for the evaluation of spinal neuron activity in vivo, thereby providing critical insights into the mechanisms of analgesic drugs. Pain can originate from peripheral injury, central dysfunction, or proximal nerve damage, suggesting that the efficacy of analgesics may vary depending on whether their primary site of action is peripheral or central. This implies that the appropriate selection of analgesic drugs and dose may vary depending on the underlying pain condition. Also, the effective agent or dose may depend on whether the drug acts on the central nervous system or peripheral structures. To elucidate the central efficacy of analgesics, we developed a novel method enabling extracellular recording of spinal neurons following i.t. administration. While previous studies successfully assessed the effects of analgesics on pain behavior following i.t. administration, few examined spinal neuronal firing using the same administration method [7,14]. The advantage of this newly developed administration method is that it allows for the evaluation of drug distribution under the same conditions as for behavioral assessments. This enables a comparative analysis of the behavioral and electrophysiological effects of the drug.

Traditionally, the effects of drugs on the neuronal firing in the spinal cord were assessed using spinal injection and bath application techniques [10,11]. However, the i.t. administration method involves injecting the drug into the subarachnoid space situated between the arachnoid and pia mater, leading to two significant differences compared with spinal injection and bath application [15]. First, there is a difference in the site of drug distribution. Spinal injection and bath application are limited to the spinal cord, whereas i.t. administration allows for widespread diffusion throughout the entire spinal region, as well as the cauda equina and spinal nerves located in the subarachnoid space [16]. Indeed, our dye injection studies confirmed that the dye reaches the cauda equina (Figure 1). Second, the drug concentration may differ between methods. In i.t. administration, the drug is diluted by the CSF, whereas spinal injection and bath application involve direct application of the drug solution into the spinal cord, resulting in different drug concentrations in the spinal cord. To address this issue, we developed a new i.t. method for evaluating spinal neuronal activity. In this method, since the drug is injected into the subarachnoid space in the same way as general i.t. administration, it is assumed that the intratissue concentration and the area of drug distribution will be close to that of general i.t. administration. In fact, we confirmed that the effect of suppressing spinal nerve firing correlated with the analgesic effect of lidocaine i.t. administration. This method can eliminate the differences due to different administration methods and allow a clearer understanding of the mechanisms underlying analgesic efficacy. We anticipate that this method can be applied to disease models such as cauda equina compression (CEC) [17]. The cauda equina is located near the spinal cord and is a critical tissue often affected in conditions such as spinal stenosis while playing a significant role in pain transmission [18]. Therefore, the efficacy of the drug in the CEC model may vary depending on its blood–brain barrier (BBB) permeability including the ability to distribute to the cauda equina, in the presence or absence of central action. By evaluating both the pharmacological effects and their mechanisms within central tissues, this method can facilitate drug profiling in conditions involving damage to regions adjacent to central structures.

Intrathecal catheter placement is frequently used in both experimental models and clinical settings, offering reproducibility, precise targeting, and reduced human error [19,20,21]. However, to date, there have been few reports of in vivo electrophysiological studies employing i.t. administration via catheterization. In the present study, we used acute i.t. administration rather than catheterization to evaluate the immediate effects of the drug. Because lidocaine acts acutely and transiently as demonstrated in Figure 4, it was essential to perform rapid administration following laminectomy and to begin neuronal recordings without delay. In the future, catheterization may be considered for studies requiring chronic or repeated drug administration.

In our evaluation of spinal neuronal firing, we observed temporal changes for up to 105 min following i.t. administration of lidocaine. Furthermore, we previously conducted similar electrophysiological techniques to assess spinal neuronal activity over extended periods [22]. These results suggest that our method allows evaluation of drug efficacy over several hours and enables observation of drug washout. Although we have not conducted recordings over multiple days post-injection, the ability to track time-dependent changes offers significant advantages for drug profiling.

However, it is important to note several limitations associated with our method and the interpretation of the results. First, it has been reported that i.t. administration can also lead to drug distribution to the dorsal root ganglia (DRG), which means that the observed effects may not entirely be attributed to central action. The influence of the drug on the DRG might suppress peripheral nerve conduction, leading to the observed reduction in spinal neuronal firing. To accurately demonstrate the central effects of the drug, it is crucial not only to evaluate the i.t. administration, but also to incorporate assessments of other administration routes, such as peripheral nerve or intraganglionic injections, alongside evaluations of both spinal and peripheral nerve activity. Second, we were unable to record baseline (pre-treatment) neuronal activity due to the need to maintain membrane integrity during i.t. administration. As a result, we had to rely on comparisons between vehicle-treated and drug-treated groups rather than pre- and post-treatment data from the same neurons.

Despite these limitations, our approach enables the simultaneous assessment of drug effects on both behavioral and spinal neuronal responses, providing a powerful tool for elucidating analgesic mechanisms. Future studies using this methodology could extend the analysis to other analgesic agents, enhancing our understanding of their mechanisms of action. Moreover, combining this approach with disease models that feature central or central proximal nerve pathology—such as the CEC model—could further enhance our understanding of chronic pain mechanisms and the estimation of the clinical efficacy of analgesics in conditions characterized by central damage.

## 5. Conclusions

In conclusion, our developed method has the potential to shed light on the central effects of analgesics and their underlying mechanisms. Understanding the specific mechanisms of analgesics may result in appropriate drug and dosage selection to manage various pain conditions.

## Data Availability

The original contributions presented in this study are included in the article, and further inquiries can be directed to the corresponding author.

## Abbreviations

The following abbreviations are used in this manuscript:

i.t.	Intrathecal
i.p.	Intraperitoneal
CSF	Cerebrospinal fluid
vFF	von Frey filaments
AP	Action potential
CEC	Cauda equina compression
BBB	Blood–brain barrier
DRG	Dorsal root ganglia
